# Genome-Wide Identification and Characterization of the WRKY Gene Family in *Cucurbita maxima*

**DOI:** 10.3390/genes14112030

**Published:** 2023-10-31

**Authors:** Qin Zhou, Ziqing Guo, Xiaojun Zhou, Lei Zhou, Duanhua Wang, Kailiang Bo, Pu Zhu

**Affiliations:** 1 Jinhua Academy of Agricultural Sciences, Jinhua 321000, China; 2 Anhui Provincial Key Laboratory of Melons and Vegetables Germplasm Resource Innovation and Intelligent Technology, Hefei 230031, China; 3 Hunan Vegetable Research Institute, Changsha 410125, China; 4 State Key Laboratory of Vegetable Biobreeding, Institute of Vegetables and Flowers, Chinese Academy of Agricultural Sciences, Beijing 100081, China

**Keywords:** *Cucurbita maxima*, WRKY, transcription factor, biotic stress, CMV

## Abstract

In higher plants, WRKY transcription factors are broadly involved in a variety of life activities and play an important role in both biotic and abiotic stress responses. However, little is known about the functions of *WRKY* genes in the popular species, such as *Cucurbita maxima* (pumpkin), which is planted worldwide. In the present study, 102 *CmWRKY* genes were identified in the *C. maxima* genome. Chromosome location, multiple sequence alignment, phylogenetic analysis, and synteny analysis of the CmWRKYs were performed. Notably, we found that silencing *CmWRKY22* promoted cucumber mosaic virus (CMV) infection, whereas overexpression of *CmWRKY22* inhibited the CMV infection. Subsequently, an electrophoretic mobility shift assay (EMSA) confirmed that *CmWRKY22* was able to bind to the W-box at the promoter of *CmPR1b*, which is a responsive gene of the salicylic acid (SA) signaling pathway. In summary, this study has provided a foundation for the antiviral functions of WRKY transcription factors in *C. maxima*.

## 1. Introduction

Transcription factors (TFs) play an important role in plants, as evidenced by the sequence-specific DNA binding and the activation or repression of downstream target gene expression [1,2]. Such properties are not limited to higher plants but are present in other living organisms. Because of its important biological functions, TFs are also commonly used as targets for molecular breeding, a process that involves not only the control of gene expression but also a large number of cellular processes [3,4]. Considering the WRKY family being one of the largest classes of transcription factor families in plants, it contains multiple family members, such as basic helix–loop–helix (bHLH), ethylene responsive factor (ERF), CUC2 (cup-shaped cotyledon) (NAC), basic leucine zipper (bZIP), myeloblastosis (MYB), NAM (no apical meristem), ATAF1/2, and C2H2 families [5]. The potential of the WRKY family to participate in a diversity of biological processes (e.g., stress tolerance, growth and development) has recently attracted considerable attention [6,7].

Generally, WRKY TFs contain one or two highly conserved domains of approximately 60 amino acids in size. They have a conserved seven amino acid–peptide (WRKYGQK) signature at the N-terminus of the protein, and a zinc finger motif (C_2_H_C_ or C_2_H_2_) at the C-terminus of the protein [8]. This highly conserved domain can bind to the W-box region of downstream target genes to regulate their expression. For example, the conserved WRKYGQK domain in *AtWRKY4* has been found to be directly involved in DNA binding, by using nuclear magnetic resonance [9,10]. Considering that research on *Arabidopsis thaliana* WRKY genes is relatively early and comprehensive, the existing classification of the WRKY gene for other species is also guided by the classification guidelines for the *Arabidopsis* WRKY genes. As a matter of fact, *WRKY* genes can be divided into three groups according to their sequence characteristics and motifs: Groups I, II and III [8]. Group II is further divided into five subgroups: IIa, IIb, IIc, IId, and IIe [8]. The difference between Group I, and Groups II and III is that the *WRKY* genes in Group I contain two WRKY domains, whereas those in Groups II and III contain only one WRKY domain. The difference between Groups II and III is that they have different zinc finger motifs, C2H2 (C-X_4-5_-C-X_22-23_-H-X_1_-H) and C2HC (C-X_7_-C-X_23_-H-X_1_-C), respectively [11]. Recently, several *WRKY* genes with important functional significance were characterized in different WRKY groups. For example, *GhWRKY25*, a Group I family member cloned from cotton, was found to have a lower expression level under abiotic stresses, whereas overexpression of *GhWRKY25* could promote resistance to the pathogen *Botrytis cinerea* and tolerance to drought in *Nicotiana benthamiana* [12]. Following exposure to external environmental stress factors, the expression levels of *VfWRKY1* and *VfWRKY2* in *Vicia faba* L. [13] and *CsWRKY2* in *Camellia sinensis* [14] become abnormal. A similar situation was observed in Groups II and III. For example, four *WRKY* genes (*AtWRKY6*, *AtWRKY11*, *AtWRKY54*, and *AtWRKY70*) identified in *A. thaliana*, *GhWRKY17* identified in *Gossypium hirsutum*, and *MtWRKY76* identified in *Medicago truncatula* could enhance leaf senescence, and drought and salt tolerances [14,15,16,17]. In addition, the abnormal expression of *WRKY* genes can be induced by hormone stimulation in plants [18]. Taken together, *WRKY* genes elicit a wide range of responses when plants are subjected to external environmental stress, indicating the functional diversity of *WRKY* genes.

Pumpkin (*C. maxima*) belongs to the *Cucurbitaceae* family. It has a high yield and is an important cultivar that has brought significant economic benefits to the world [19]. It is used in a variety of ways, such as soups, purees, jams, and pies, for human consumption [20]. WRKY TFs have been identified in several species but have been less studied in the *Cucurbitaceae* family. In the present study, we identified 102 *CmWRKY* genes by searching the *C. maxima* genome. We clarified the classification of *CmWRKY* genes by the comparison with the WRKY family of *A. thaliana* (*AtWRKYs*). Multiple sequence alignments of amino acids, conserved motifs, gene structure, and synteny analysis were also performed in this study. Interestingly, the WRKY gene *CmWRKY22* was found to play a positive regulatory role in plant resistance to cucumber mosaic virus (CMV) infection. Electrophoretic mobility shift assay (EMSA) experiments indicated that *CmWRKY22* may bind to the W-BOX of the *CmPR1b* promoter region to exert its antiviral effects. These studies provide greater insight into the participation of plant WRKY TFs in virus–host interactions.

## 2. Materials and Methods

### 2.1. Identification of WRKY Proteins in C. maxima

In order to obtain the full set of *C. maxima WRKY* genes, we downloaded the *C. maxima* genome, including gene prediction information and predicted protein sequences from the Cucurbit Genomics Database (http://cucurbitgenomics.org/, accessed on 15 July 2022). The WRKY protein sequences of *A. thaliana* was downloaded from PlantTFDB (http://planttfdb.gao-lab.org, accessed on 19 July 2022). The Hidden Markov Model (HMM) profile of the WRKY domain (PF03106) was downloaded from the Pfam database (http://pfam.xfam.org/, accessed on 30 July 2022), and the HMMER software (http://hmmer.org/, accessed on 30 July 2022) was used to search for WRKY proteins in the full *C. maxima* protein. We used the Batch Web CD-Search Tool in NCBI (https://www.ncbi.nlm.nih.gov/Structure/bwrpsb/bwrpsb.cgi, accessed on 1 August 2022) and the Search Pfam tool in the Pfam database to verify the presence of the WRKY domain in the candidate genes. Genes without intact WRKY domains were excluded. We then renamed CmWRKYs according to their chromosomal location. The Molecular weight (MW), Open reading frame (ORF), Aliphatic index (Ai), Isoelectric point (PI), and Grand average of hydropathicity (GRAVY) were were computed for WRKY proteins in *C*. *maxima* using the Expasy online software (https://web.expasy.org/protparam/, accessed on 1 August 2022). Plant-mPLoc (http://www.csbio.sjtu.edu.cn/bioinf/plant-multi/, accessed on 25 July 2022) was used to predict the subcellular localization of CmWRKY proteins.

### 2.2. Multiple Sequence Alignment and Phylogenetic Analysis

All 102 predicted CmWRKY proteins with amino acids spanning the WRKY core domain were used to create multiple alignments using MUSCLE [21]. A phylogenetic tree was constructed based on the N-terminal WRKY domain of each CmWRKY protein alignment using the maximum likelihood in IQ-TREE [22], and with 1000 ultrafast bootstraps. The groups and subgroups of the WRKY family in *C. maxima* were divided according to the previously reported classification of the WRKY family in *A. thaliana*.

### 2.3. Conserved Motifs, Protein Structure, Synteny Analysis, and Chromosomal Location

To reveal the conserved motif of all CmWRKY proteins, we used the MEME software (https://meme-suite.org/meme/tools/meme, accessed on accessed on 1 August 2022). The motif location, gene structure information (such as introns and exons) and chromosomal localization of the *CmWRKY* genes were then visualized using TBtools [23]. Likewise, we performed a synteny analysis using MCSCcanX and TBtools to study the synteny events of *C. maxima* [24]. At the same time, to determine the chromosomal location of the *CmWRKY* genes, we downloaded the genes from the Cucurbit Genomics Database (http://cucurbitgenomics.org/, accessed on 15 July 2022), mapped them to the *C. maxima* chromosomes, and renamed these WRKY genes *CmWRKY1-102* according to the position of the gene on the chromosome using TBtools [23]. In addition, previous studies have shown that *C. maxima* has 20 chromosomes [25]. They were named chr1-20 in this study.

### 2.4. Plant Growth and Virus Inoculation

*C. maxima* and *N. benthamiana* seeds were germinated in plots containing vermiculite and substrate (W/W, 1:3). The temperature of the greenhouse was maintained at 25 °C with 60% humidity, and in a 16 h/8 h (light/dark) cycle. Three-week-old *C. maxima* plants were used for agrobacterial infiltration of TRV RNA1 and RNA2 for the TRV assay. For virus inoculation, agrobacterium strain harboring CMV RNA1, RNA2 and RNA3 were mixed at 1:1:1 ratio and co-transformed into *N. benthamiana*, P19 as the silencing suppressor was also co-infiltrated at an OD600 of 0.5–0.8. After inoculation, the leaves were inoculated in greenhouse for 3–5 days and then collected for quantitative RT-PCR analysis.

### 2.5. RNA Isolation and Quantitative RT-PCR Analysis

To examine gene expression during biotic stress, total RNA extraction and reverse transcription were conducted according to the manual protocol. Briefly, 1.5 μg total RNA was reverse transcribed under the following conditions: 5 min at 25 °C, 45 min at 37 ℃, 5 s at 8 °C, 5 min at 4 °C. The cDNA was used for qRT-PCR. Specific primers were designed using the Primer-BLAST software (https://blast.ncbi.nlm.nih.gov/Blast.cgi, accessed on 30 November 2022). Quantitative RT-PCR analysis was carried out using AceQ qPCR SYBR Green Master Mix (Vazyme, Nanjing, China) and the ABI Q5 Sequence Detection System (Applied Biosystems, Foster City, CA, USA) software 1.4.1. All the data were calculated using the 2^−ΔΔCT^ method [26]. At least three biological replicates were used for the analysis. The *β-actin* gene and *N. benthamiana* actin gene (GenBank No. JQ256516) were used as internal reference genes in the analysis.

### 2.6. Plasmid Construction and Agrobacterium Transformation

For the TRV-based virus-induced gene silencing assay, the full coding sequence (CDS) of *CmWRKY22* was amplified with the specific primer *CmWRKY22*. The product was digested into the TRV vector after digestion with the corresponding restriction enzymes *BamHⅠ/SmaⅠ* (Thermo Fisher Scientific, Beijing, China) to generate TRV RNA2 (CmWRKY22). TRV RNA2 (CmWRKY22) plasmid was then individually transformed into *Agrobacterium Tumefaciens* GV3101. In addition, the coding sequence of CmWRKY22 was amplified, cloned and inserted into expression vectors pGWB5C to generate pGWB5C: CmWRKY22-GFP and transformed into *Agrobacterium Tumefaciens* GV3101.

### 2.7. Electrophoretic Mobility Shift Assay (EMSA)

The ability of *CmWRKY22* to bind *CmPR1b* was analyzed using EMSA. The W-box of the *CmPR1b* promoter was used to synthesize biotin end labels. Unlabeled (competitor) and labeled (mut competitor) W-box oligonucleotides were used as controls. The EMSA kit was purchased from Thermo Scientific (Waltham, MA, USA) and was used following the manufacturer’s protocol.

## 3. Results

### 3.1. Identification of the WRKY Gene Family in C. maxima

**Figure 1:**
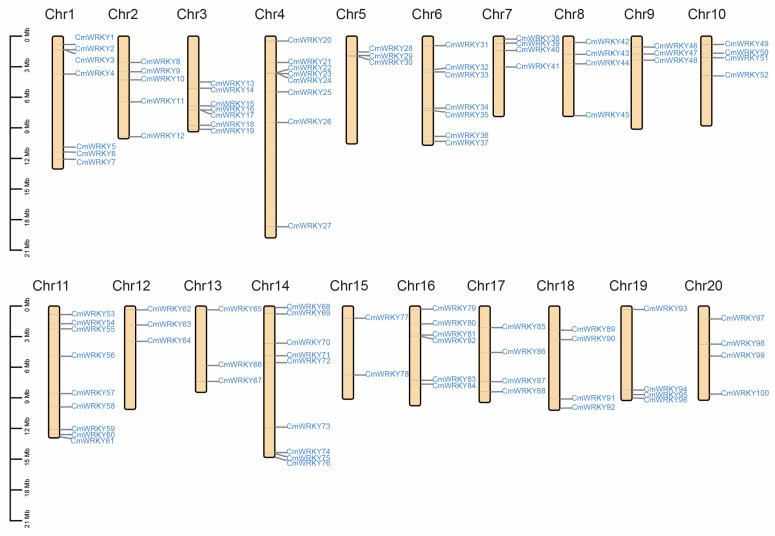
Mapping of the WRKY gene family on *C. maxima* genome. The size of a chromosome is indicated by its relative length. The 100 putative WRKY genes were renamed from *CmWRKY1* to *CmWRKY100* according to their chromosome location. Two putative WRKY genes (*CmWRKY101* and *CmWRKY102*) could not be localized on a specific chromosome.

A total of 102 genes encoding putative WRKY proteins were identified in the *C. maxima* genome. Among them, 100 *WRKY* genes were mapped on chromosomes 1–20 and were named *CmWRKY1* to *CmWRKY100* according to their chromosomal locations and two WRKY genes could not be mapped to any chromosome and were named *CmWRKY* 101 and 102 (Figure 1). Furthermore, chromosomes 11 and 14 had the largest number of *CmWRKY* genes (9, 9%), whereas chromosome 15 had the smallest (2, 2%). Seven chromosomes had four genes localized on them, which implies that genetic variations in *CmWRKY* genes might exist in the evolution process. Several parameters of *CmWRKY* genes are listed in Table S1, including the deduced protein length, MW, PI, predicted subcellular localization, AI, and GRAVY. The deduced lengths of the CmWRKY proteins range from 145 (*CmWRKY64*) to 1305 amino acids (*CmWRKY27*), whereas the MW range from 16.79 kDa to 145.74 kDa. In addition, the isoelectric point ranges from 4.78 (*CmWRKY78*) to 9.8 (*CmWRKY50*), whereas the AI range from 43.49 (*CmWRKY39*) to 76.95 (*CmWRKY41*). The different features of these proteins indicate their functional diversities.

### 3.2. Phylogenetic Analysis of CmWRKY Gene

**Figure 2:**
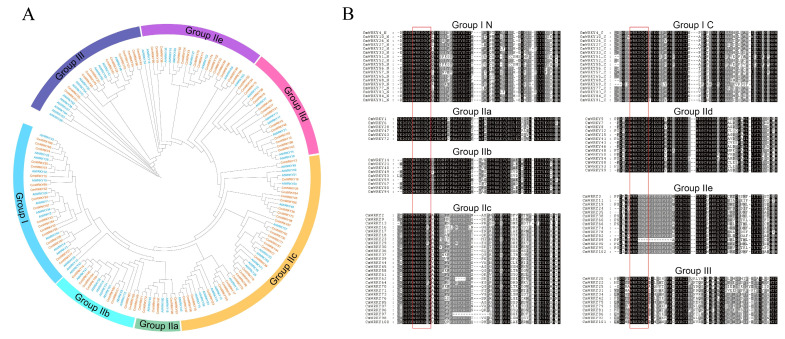
Unrooted Phylogenetic tree and multiple sequence alignments of *CmWRKY* domains. (**A**) The phylogenetic tree was constructed using maximum likelihood in IQ-TREE based on N-terminal WRKY domains. Orange represents the WRKY gene in *C. maxima* and blue represents the WRKY gene in *A. thaliana*. The different colors on the outer ring represent different groups. (**B**) The N-terminal WRKY domains and C-terminal WRKY domains of Group I WRKYs are shown by the “Group IN” or “Group IC”, respectively. The conserved 7-peptide amino acids are marked with a red box, and the alignments were performed using MUSCLE.

To examine the phylogenetic relationship of the *WRKY* gene in the *C. maxima* family, 102 *CmWRKY* genes from *C. maxima* and 75 *AtWRKY* genes from *A. thaliana* were selected for phylogenetic analysis. A previous study has shown that the *WRKY* gene family is highly conserved in monocots and eudicots and is divided into three groups [27]. The WRKY proteins in *C. maxima* were classified into three groups: I, II, and III. The largest of these groups, Group II, contained 72 CmWRKY proteins and accounted for 70% of all the CmWRKY proteins in *C. maxima*. Groups I and III contained 18 and 12 CmWRKY proteins, accounting for 18% and 12% of all CmWRKY proteins in *C. maxima*, respectively. In addition, Group II was further divided into five subgroups (IIa, IIb, IIc, IId, and IIe) containing 6, 9, 28, 14, and 15 CmWRKY proteins, respectively (Figure 2A). Similar to AtWRKYs, most CmWRKY proteins are located in Group IIc. Group I contains two conserved WRKY domains, one with the zinc finger motifs C-X_4_-C-X_22_-H-X-H in the N-terminus and the other with the X_4_-C-X_23_-H-X-H in the C-termini (Table S1, Figure 2B), while Groups II and III contain only one WRKY domain. Notably, among the five subgroups of Group II, the zinc finger motifs of subgroup IIc are different from those of the other subgroups. Specifically, the motifs of IIc are C-X_4_-C-X_23_-H-X-H, and those of IIa, IIb, IId, and IIe are C-X_5_-C-X_23_-H-X-H (Table S1, Figure 2B). Moreover, the motif of Group III is C-X_7_-C-X_23_-H-X-H (Table S1, Figure 2B).

### 3.3. Conserved Motifs and Gene Structure of CmWRKY Proteins

**Figure 3:**
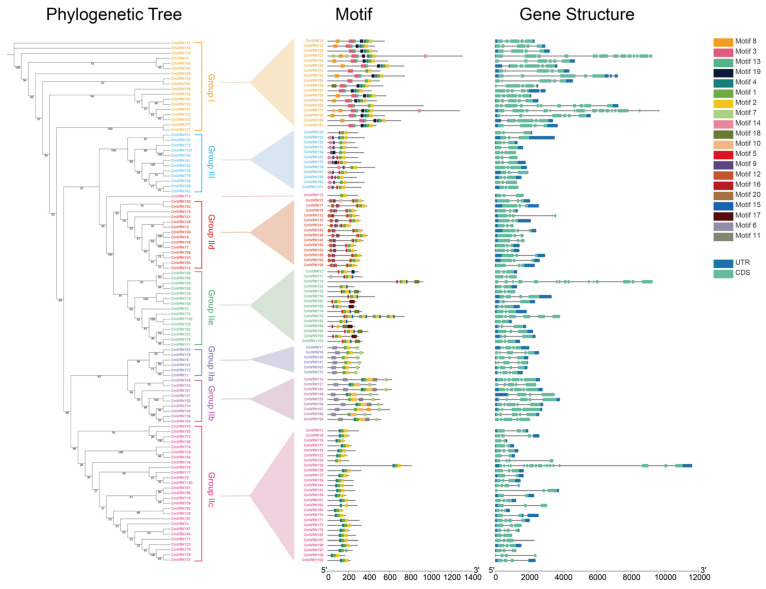
Phylogenetic relationship, motif compositions, and intron–exon structures. The unrooted phylogenetic tree was constructed using IQ-TREE by the maximum likelihood method with 1000 ultrafast bootstrap replicates. The middle figure displays the motif compositions of CmWRKY proteins as determined by MEME software 5.5.4 analysis, with various colored boxes serving as representations. An intron–exon structure of the 102 *CmWRKY* genes is shown in the right panel. The scale at the bottom can be used to assess length.

To further understand the conservation of CmWRKYs, we surveyed the gene structure and conserved motif of all *CmWRKY* genes identified using the Multiple EM for Motif Elicitation (MEME) software 5.5.4. Eleven motifs, named 1–11, were identified (Figure 3). The number of conserved motifs for each *CmWRKY* gene ranged from two (*CmWRKY13* and *CmWRKY62*) to nine (*CmWRKY27* and *CmWRKY77*) (Figure 3). All *CmWRKYs* contained motif 1 and motif 2. The conserved motifs of *CmWRKYs* categorized into the same group or subgroup had highly similar compositions. For example, in subgroup IIc, most *CmWRKYs* are composed of motifs 4-1-2, except for *CmWRKY97* and *CmWRKY62*, which are composed of 4-1-13 and 4-2, respectively. At the same time, Group IIc has the lowest number of motifs. Subgroup IIa has the same motif composition (6-1-2-7). Groups I and IId has rich motif compositions, and all groups contain motifs 1 and 2. In contrast, motifs 12 and 15 are only present in subgroups IId and IIe, respectively. Motif 6 was found only in subgroups IIa and IIb, and motif 3 was only found in Group I.

The structural features and evolution of *CmWRKY* genes were further analyzed based on the intron/exon distribution and intron number. The number of introns in the CmWRKY family ranges from one to 16 (Figure 3). A total of 46 *CmWRKY* genes contain two introns, which is the largest proportion, 18 *CmWRKY* genes contain four introns, 16 *CmWRKY* genes contain three introns, seven *CmWRKY* genes contain five introns, and two *CmWRKY* genes contain 14 introns. In addition, nine *CmWRKY* genes contain only one intron, and only one *CmWRKY* gene contains 6, 10, 12, and 16 introns, respectively. *CmWRKY* genes, categorized into the same group and subgroup, have similar intron numbers. For example, in subgroup IId, only one *CmWRKY* gene had three introns, while the others had two. Most *CmWRKY* genes in subgroups IIc, IIe, and III also had two introns. In contrast, *CmWRKY* genes in Group I, and subgroups IIa and IIb all have more than two introns, some have four or five introns, and others have even more than six introns.

Hence, the similar motif and gene structure of proteins in the same group or subgroups suggests that these proteins may have similarities in the role of these proteins.

### 3.4. Synteny Analysis of CmWRKYs

**Figure 4:**
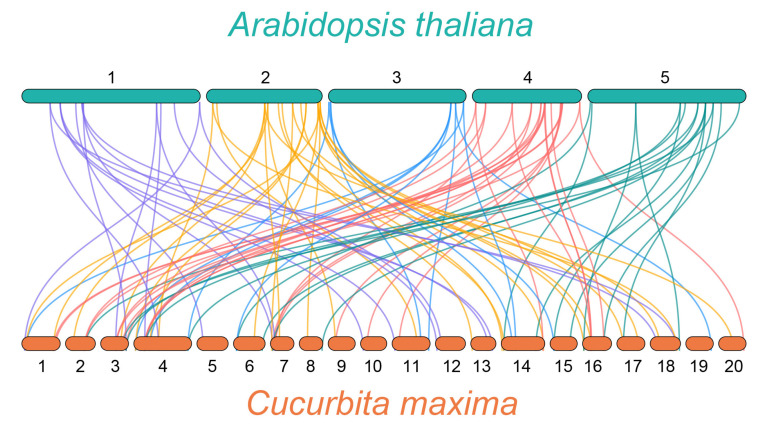
Synteny analyses between the *WRKY* genes of *C. maxima* and *A. thaliana*. The collinear gene pair with *A. thaliana* genes are highlighted with different color lines.

As a model plant, the WRKY family of *A. thaliana* has been systematically studied for many years [28]. Thus, to understand the evolution of the *C. maxima* WRKY family members, a synteny analysis was performed between *C. maxima* and *A. thaliana* (Figure 4, Table S2). The differently colored lines in the background highlight syntenic *WRKY* gene pairs with different chromosomes of *C. maxima* and *A. thaliana*. A total of 90 pairs of syntenic relationships were identified, including 48 *AtWRKY* genes and 63 *CmWRKY* genes. Among these *WRKY* genes, five *CmWRKY* genes (*CmWRKY11*, *CmWRKY14*, *CmWRKY18*, *CmWRKY39*, and *CmWRKY40*) and three *AtWRKY* genes (*AtWRKY61*, *AtWRKY70*, and *AtWRKY29*) were found to be associated with three synteny events. Three *AtWRKY* genes (*AtWRKY15*, *AtWRKY23*, and *AtWRKY48*) were associated with four synteny events, and one *AtWRKY* gene (*AtWRKY46*) was associated with five synteny events. Two synteny events accounted for the largest proportion of 90 pairs of synteny relations.

### 3.5. CmWRKY22 Negatively Regulated CMV Infection

**Figure 5:**
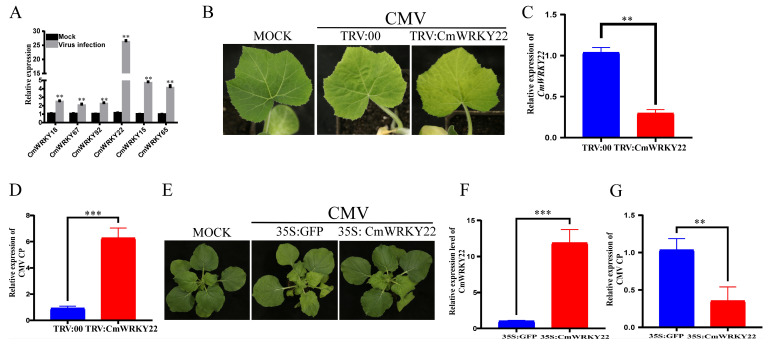
*CmWRKY22* plays a negative role in CMV infection. (**A**) The relative expression level of six *CmWRKY* genes after CMV infection. (**B**) Tobacco Rattle Virus (TRV)-based virus-induced gene silencing of *CmWRKY22* promoted CMV infection and exhibits more mosaic leaf symptoms. (**C**) The relative expression level of CmWRKY22 in TRV:00 and TRV: CmWRKY22 plants. (**D**) The relative expression level of CMV CP in C. maxima plants (TRV: CmWRKY22) compared with control (TRV:00) plants. (**E**) *CmWRKY22* inhibited CMV infection in N. benthamiana. (**F**) The relative expression level of CmWRKY22 in 35S: GFP and 35S: CmWRKY22 in N. benthamiana. (**G**) The relative expression of CMV CP in 35S: CmWRKY22 compared with control plants. These qRT-PCR results suggested that *CmWRKY22* plays a negative role in CMV infection. The asterisks represent significant differences of treatment group and control group (Student’s *t*-test, ‘***’, *p* < 0.001 ‘**’, *p* < 0.01), all data were analyzed using 2^−ΔΔCT^ method.

WRKY TFs are involved in plant resistance to viruses The initial homology analysis in our study indicated that six *CmWRKY* genes might be involved in the process of virus resistance using homology analysis [29,30,31,32,33,34] (Table S3). To confirm the virus resistance function of these genes, the relative gene expression levels were analyzed after CMV infection. The results showed that all six *CmWRKY* genes were induced following CMV infection, with *CmWRKY22* showing the highest expression (Figure 5A). Therefore, we focused our investigation on the role of *CmWRKY22* in CMV infection. We used tobacco rattle virus (TRV)-based virus-induced gene silencing in *C. maxima* plants. Notably, the full coding sequence of *CmWRKY22* was cloned into TRV RNA2 to generate TRV: CmWRKY22 and co-inoculated with TRV RNA1. Compared with the control plants, TRV: CmWRKY22 exhibited more severe viral disease symptoms on leaves after CMV infection (Figure 5B). Meanwhile, the relative expression level of *CmWRKY22* in TRV:00 (control) was significantly higher, while the expression level of CMV Coat Protein (CP) was lower (Figure 5C,D). In addition, we examined the *CmWRKY22* and CMV CP expression levels in *CmWRKY22* transient overexpression plants. As shown in (Figure 5E–G), the symptoms and virus accumulation were significantly lower in the *CmWRKY22* overexpression plants than in the control plants. These results indicate that *CmWRKY22* may have a negative effect on CMV infection.

### 3.6. CmWRKY22 Regulated the Expression of Salicylic Acid (SA)-Responsive Gene PR1b in Plants

**Figure 6:**
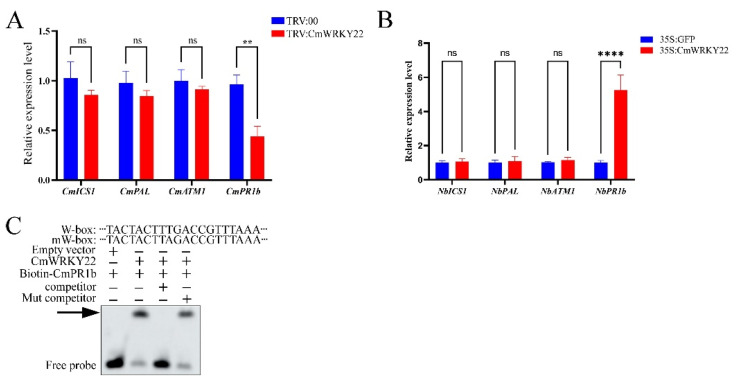
*CmPR1b* was significantly induced and *CmWRKY22* could regulated the expression of SA-responsive gene *CmPR1b*. (**A**) Relative expression level of SA biosynthesis and responsive genes between TRV:00 and TRV: CmWRKY22. (**B**) Relative expression level of SA biosynthesis and responsive genes. The asterisks in A and B represent significant differences of treatment group and control group (Student’s *t*-test, ‘**’, *p* < 0.01, ‘****’, *p* < 0.0001, ns, no significance), all data were analyzed using 2^−ΔΔCT^ method. (**C**) EMSA results confirmed the *CmWRKY22* binds to the W-box of *CmPR1b* promoter. The arrow indicated the binding of *CmWRKY22* to the biotin-labled W-box promoter. The “+” and “−” indicated the correspond component.

Since SA plays a crucial role in plant resistance to pathogens and viral infections [35,36], we have further investigated if *CmWRKY22* is involved in the SA signaling pathway. Using the TRV assay, we found that the expression of SA synthesis-related genes (*CmICS1*, *CmPAL*, and *CmATM1*) was not significantly different between TRV:00 and TRV:CmWRKY22, whereas the expression of SA-responsive genes (*pathogenesis-related proteins b, PR1b*) was significantly higher (Figure 6A). In addition, only the expression of *NbPR1b* was significantly higher in *CmWRKY22* transient overexpression plants. (Figure 6B). WRKY TFs were known to be able to bind to the W-box in the promoter region of a gene to regulate its expression. Thus, we performed an EMSA analysis of *CmWRKY22* with the W-box region of the *CmPR1b* promoter, and the results showed that *CmWRKY22* was able to bind the W-box motif of the *CmPR1b* promoter (Figure 6C).

## 4. Discussion

The WRKY TF family is engaged fundamentally in the entire plant growth and development process and plays a pivotal role in the exposure of both biotic and abiotic stresses [7,37]. It has been studied extensively in sereral monocot and dicot species, including *A. thaliana* [38], Rice [38] and Wheat [39]. However, the identification of WRKY TFs in the *Cucurbitaceae* family pales in comparison to other large families, such as only 63 WRKY genes were identified in *Citrullus lanatus* [40] and 55 WRKY genes in *Cucumis sativus* [41]. However, we identified 102 WRKY genes in the *C. maxima*, which is much more than the above *C. lanatus* and *C. sativus*, suggesting that the WRKY TFs in the *Cucurbitaceae* family still has great potential for exploitation.

Gene duplication has a vital part in biological evolution as it helps to increase genetic and functional diversity [42,43,44] and can result in the expansion of WRKY gene family members [42]. In this study, we identified 102 WRKY genes in the *C. maxima* genome that were distributed in a diverse manner across 20 chromosomes (Figure 1) and were extremely similar in their number to *A. thaliana* (102). However, the number of WRKY genes is significantly larger as compared to the number of WRKY genes on some other species, such as *Isatis indigotica* (64) [45] and *Salvia miltiorrhiza* (61) [46]. Therefore, we speculate that gene duplication events may also occur in *C. maxima*. A syntenic relationship analysis based on CmWRKY family with AtWRKY family has shown a total of 90 pairs of syntenic relationships between *C. maxima* and *A. thaliana*, suggesting that the evolution of *C. maxima* and *A. thaliana* are quite closely related.

CMV infection is a major plant viral disease that affects a variety of plants and causes severe yield losses. WRKY transcription factors are involved in various biological processes in plants and play a broad-spectrum regulatory role in plant defense responses [36,47,48]. Therefore, to gain a better understanding of the role of WRKY transcription in CMV infection of *C. maxima*, we have selected several genes that may be involved in the response to CMV infection. These are homologs of genes that have been reported to play important roles in viral infection in other species, including *A. thaliana* [29,30,31], *Solanum lycopersicum* [32], *G. hirsutum* [33], and *Capsicum annuum* [34]. Six candidate genes were identified, among which *CmWRKY22* showed the highest expression, reaching >25-fold higher levels after CMV infection. Therefore, we conducted subsequent experiments using *CmWRKY22* as a target. The other five genes, which may also be involved in the process of CMV–host interactions, will be the targets of our future studies.

*C. maxima* WRKY TFs have been divided into three groups: I, II, and III, while CmWRKY22 belongs to Group III. Several studies have been conducted on Group III members in different species, including *A. thaliana* [49], *rice* [50,51], *grape* [50], and *S. lycopersicum* [31]. Prior studies have shown that Group III members contribute to the pathogen defense efforts, for example by participating in the SA pathway in plants [31,49]. It has been reported that SA also plays an important role in plant resistance to biotic stress, including viruses and pathogens [36,52,53,54,55]. Meanwhile, *AtWRKY22* can regulate SA and jasmonic acid (JA) signaling [56], which leads us to speculate that *CmWRKY22* is also involved in this process.

Therefore, we used TRV-based, virus-induced gene silencing and overexpression to explore the functions of *CmWRKY22*. As shown in Figure 5, *CmWRKY22* plays a negative regulatory function in CMV infection. Then, we analyzed the expression levels of three SA synthesis-related genes and one SA-responsive gene in the TRV assay and overexpression plants. The results showed that the SA-responsive gene *PR1b* was significantly upregulated (Figure 6A,B). As one of the largest TF families, the WRKY TF family can bind to the W-box (TTGACC/T) of gene promoters and regulate gene expression [57,58]. The EMSA also showed that *CmWRKY22* could bind to the promoter of *CmPR1b* (Figure 6C). Therefore, we speculate that *CmWRKY22* may bind to the W-box of *CmPR1b* to regulate its expression and, thus, protect against CMV infection.

## 5. Conclusions

One-hundred-and-two WRKY genes were identified in *C. maxima*. Their chromosomes’ location, phylogenetic relationship, conserved motifs, and gene structure were examined. Moving a step further, we identified a gene, *CmWRKY22*, that plays a positive regulatory role in plant resistance to cucumber mosaic virus (CMV) infection, and subsequently confirmed the ability of *CmWRKY22* to bind to the promoter region of the SA-responsive gene, *CmPR1b*, via EMSA experiments.

## Data Availability

The data that support this study are available in the article.

## Supplementary Materials

The following supporting information can be downloaded at: https://www.mdpi.com/article/10.3390/genes14112030/s1, Table S1. Identified WRKY genes in *C. maxima*. Abbreviations: Ai, aliphatic index; Chr, chromosome numbers; GRAVY, grand average of hydropathicity; MW, molecular weight; NA, not Available; ORF, opening reading frame; pI, isoelectric point; UN, unknow chromosome. Table S2. The synteny regions between *C. maxima* WRKYgenes. Table S3. List of *CmWRKY* genes may be involved in the process of virus resistance.
